# A Field Analysis of Standardizing Place-Based Indicators.

**DOI:** 10.23889/ijpds.v7i3.2035

**Published:** 2022-08-25

**Authors:** Isabel Youngs, Joseph Scariano

**Affiliations:** 1 Georgetown University

There is a lack of evidence that place-based indicators effectively measure predictors and outcomes at appropriate geographies and temporal scales. For example, shifts in collection method or definitions can result in changes to components of measurement that may or may not be concomitant with changes in the populations being measured. The Massive Data Institute is currently conducting a landscape analysis of place-based indicators and is in the process of reaching out to hundreds of experts in order to develop a framework for creating and evaluating indicators for various use cases. Standardizing the methods and components of place-based indicators can unravel relationships between measurement and differential outcomes for groups. By disentangling these relationships, the field can begin to establish best practices for equitable indicators and determine gaps or biases in measurement across groups. These standards can be replicated across various indicators, ensuring continuity of measurement across space and time.

Preliminary results reveal that one major issue with the field of place-based indicators is the definition of place that many indices and indicators use. Changes and discontinuities between census geographies decade to decade can contribute to measurement shifts or gaps in measurement for certain urban typologies, such as outlying metropolitan counties or rural communities. Additionally, these boundaries are often arbitrary and unassociated with local-level knowledge of community geography. While utilizing census boundaries can allow for data linkage across federally-created data sets, additional methods for augmenting spatial data to conduct more granular place-based analyses can assist researchers in conducting more accurate analyses for many use cases.

This presentation will dive into this issue, address some alternative methodologies and standards, and further summarize our preliminary findings and present a framework for feedback.

